# Self-Inflicted Trauma Secondary to Local Anaesthesia in Children

**DOI:** 10.1155/2017/4969484

**Published:** 2017-01-30

**Authors:** Srikrishna Vempaty, James Robbins

**Affiliations:** ^1^Oral and Maxillofacial Surgery, London North West Healthcare NHS Trust, Harrow HA13UJ, UK; ^2^Oral and Maxillofacial Surgery, Royal Cornwall Hospital, Truro, Cornwall TR13LJ, UK

## Abstract

Long acting local anaesthetics and inferior alveolar nerve block in children can cause loss of sensation and proprioception in a large area supplied by that particular nerve. Similar to the maxilla in mandible also, adequate level of anaesthesia can be achieved in the desired site of treatment by using a short acting local anaesthetic. Early return of normal sensory feedback after using short acting anaesthetics can be helpful in preventing self-harm.

## 1. Case Report

We have examined an eight-year-old girl who underwent extraction of a permanent mandibular first molar tooth on the left side under local anaesthesia and sedation in general dental practice. The following day, the child developed swelling of the lower lip and attended an out-of-hours health service with her parents. Allergic reaction to local anaesthesia or latex was suspected. The child was admitted for observation with suspected angioedema, and oral antihistamines were prescribed. The girl was then observed and discharged following a period of active monitoring. The swelling increased over the next 2 days and the child presented back to out-of-hours health service with a large sloughing ulcer measuring two centimetres in dimension. The paediatric department ruled out systemic infection and allergic reaction following blood investigations. On further enquiry into history, it was noted that the child bit the swelling following local anaesthetic administration for four to five hours as her lip felt swollen and numb. On enquiry, the dentist reconfirmed that a decayed left mandibular first molar was extracted after giving an inferior alveolar nerve block and infiltration. He maintained that 3 mL of 0.5% bupivacaine and 1 : 200000 adrenaline was administered. The child underwent the procedure under sedation as she had challenging behaviour and suffered from attention deficit hyperactivity disorder (ADHD).

**Figure 1:**
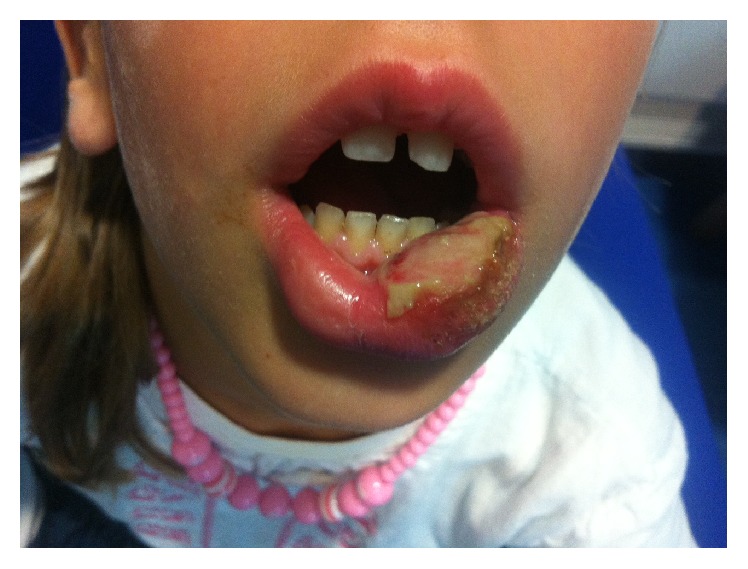
After local anaesthetic self-inflicted trauma to the lower lip.

From the history, we then realised that altered sensation on the lower lip following administration of long acting local anaesthesia made the child restless and uncomfortable. This led to the child biting on it. This gradually resulted in tissue trauma as shown in Figure 1. Parents were reassured and the child was followed up in clinic to ensure that the traumatic ulcer is healed with minimal scar.

## 2. Discussion

“The anesthetic effect of bupivacaine at a concentration of 0.5% with 1 : 200,000 epinephrine could last up to 6 hours (pulpal anaesthesia) by nerve block and between 9 and 12 hours in soft tissue.” [1] This clearly explains why the child in this case self-injured her lip. The literature also suggests that it can act for up to 3 times longer duration when compared to routinely used 2% lignocaine with 1 : 80000 epinephrine [2]. Accidental lip injury with the use of long acting local anaesthetic has been reported as one of the adverse events [3]. 2% lignocaine with 1 : 100,000 was found to achieve soft tissue anaesthesia for up to 3 to 5 hours and 60–80 minutes' pulpal anaesthesia on an average when nerve block was given [4, 5]. On the contrary, when plain lignocaine was given, soft tissue anaesthesia was up to 2 hours [5]. The use of infiltration of local anaesthetic is found to be as effective as inferior alveolar nerve block in children [6]. Sharaf in his study also found that children experienced more pain while undergoing inferior alveolar nerve block when compared to buccal infiltration. Long acting local anaesthetics also have higher neuronal toxicity [2]. Local anaesthesia with lignocaine has been shown to be sufficient for all dental procedures other than pulpotomy and extraction [7]. Perhaps a short acting local anaesthetic nerve block would have been a better choice in this case of permanent tooth extraction. Persistent altered sensation for longer duration than expected and inadvertent injury to inferior alveolar and lingual nerve also can be prevented by giving local infiltration [4].

## 3. Conclusion

Pain following tissue trauma is a physiological response. It has a protective phenomenon by which we tend to stay away from harmful activity. Children can misinterpret altered sensation due to lack of normal proprioceptive feedback. When long acting local anaesthetics are used, this uncomfortable altered sensation can lead to self-harm. So, short acting local anaesthetics without adrenaline preferably infiltrated on buccal and lingual sides can achieve the required analgesia for extractions instead of inferior alveolar nerve blocks with a long acting local anaesthetic [3]. Infiltration of local anaesthesia into the mucobuccal fold can still cause mental nerve block which may result in altered sensation over the lower lip. However, the area of disturbed sensation is still smaller when compared to inferior alveolar nerve block and if short acting anaesthetic without adrenaline is given the period of altered sensation is further reduced. It may be suggested that preventing the child from having food during this time might prevent them from smacking their lips and prevent them from exploring the vulnerable perioral tissues. Caregivers should be made aware of realistic time of action of local anaesthesia and informed about the possibility of self-inflicting injury.
